# Supplementary material for: Molecular Cloning, Characterization, and Application of a Novel Multifunctional Isoamylase (MIsA) from Myxococcus sp. Strain V11
Source: Foods. 2024 Oct 30;13(21):3481. doi: 10.3390/foods13213481 (PMC11544908; doi:10.3390/foods13213481)
Supplement: Supplementary file 1 [file foods-13-03481-s001.zip › Fig. S1.pdf]

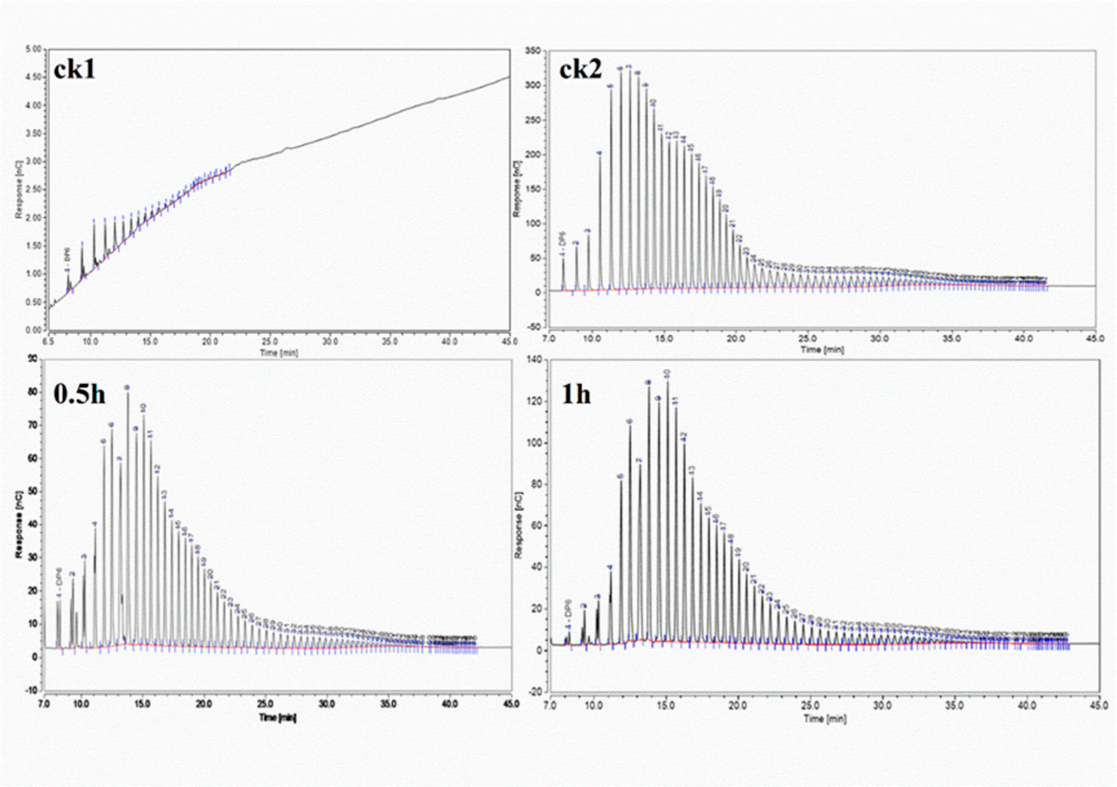

Figure S1. Analysis of the reaction products using HPAEC. Ck1 (0 h), 0.5 h and 1 h treated with MIsA. Ck2 (24 h) treated with Promozyme®D2
