# Supplementary material for: Molecular Cloning, Characterization, and Application of a Novel Multifunctional Isoamylase (MIsA) from Myxococcus sp. Strain V11
Source: Foods. 2024 Oct 30;13(21):3481. doi: 10.3390/foods13213481 (PMC11544908; doi:10.3390/foods13213481)
Supplement: Supplementary file 1 [file foods-13-03481-s001.zip › Secondary structure analysis of MIsA.pdf]

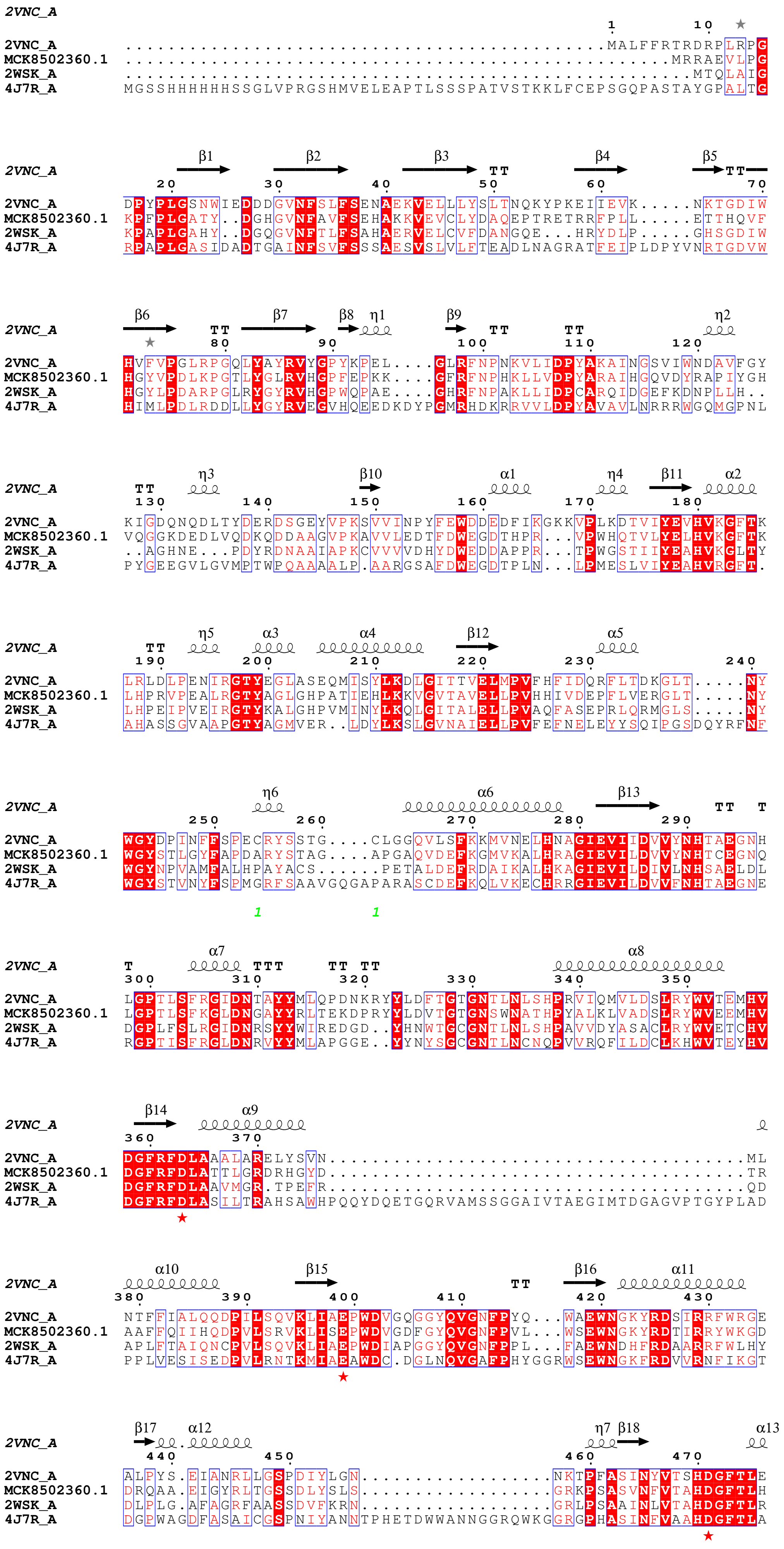

2VNC_A
1 10
2VNC_A ....MALFFRTRDRPLRPG
MCK8502360.1 ....MRRAEVLPG
2WSK_A ....MTQLAIG
4J7R_A MGSSHHHHHHSSGLVPRGSHMVELEAPTLSSSPATVSTKKLFCEPSGQPASTAYGPALTG
2VNC_A β1 β2 β3 TT β4 β5 TT
20 30 40 50 60 70
2VNC_A DPYPLGSNWIEDDDGVNFSLFSENAEKVELLLYSLTNQKYPKEIIEVK.....NKTGDIW
MCK8502360.1 KPFPLGATY..DGHGVNFAVFSEHAKKVEVCLYDAQEPTRETRRFPLL.....ETTHQVF
2WSK_A KPAPLGAHY..DGQGVNFTLFSAHAERVELCVFDANGQE...HRYDLP.....GHSGDIW
4J7R_A RPAPLGASIDADTGAINFSVFSSSAESVSLVLFTEADLNAGRATFEIPLDPYVNRTGDVW
2VNC_A β6 TT β7 β8 η1 β9 TT TT η2
80 90 100 110 120
2VNC_A HVFVPGLRPGQLYAYRVYGPYKPEL....GLRFNPNKVLIDPYAKAINGSVIWNDAVFGY
MCK8502360.1 HGYVPDLKPGTLYGLRVHGPFEPKK....GFRFNPHKLLVDPYARAIHGQVDYRAPIYGH
2WSK_A HGYLPDARPGLRYGYRVHGPWQPAE....GHRFNPAKLLIDPCARQIDGEFKDNPLLH..
4J7R_A HIMLPDLRDDLLYGYRVEGVHQEEDKDYPGMRHDKRRVVLDPYAVAVLNRRRWGQMGPNL
2VNC_A TT η3 β10 α1 η4 β11 α2
130 140 150 160 170 180
2VNC_A KIGDQNQDLTYDERDSGEYVPKSVVINPYFEWDDEDFIKGKKVPLKDTVIYEVHVKGFTK
MCK8502360.1 VQGGKDEDLVQDKQDDAAGVPKAVVLEDTFDWEGDTHPR...VPWHQTVLYELHVKGFTK
2WSK_A ..AGHNE...PDYRDNAAIAPKCVVVVDHYDWEDDAPPR...TPWGSTIIYEAHVKGLTY
4J7R_A PYGEEGVLGVMPTWPQAAAALP.AARGSAFDWEGDTPLN...LPMESLVIYEAHVRGFT.
2VNC_A TT η5 α3 α4 β12 α5
190 200 210 220 230 240
2VNC_A LRLDLPENIRGTYEGLASEQMISYLKDLGITTVELMPVFHFIDQRFLTDKGLT.....NY
MCK8502360.1 LHPRVPEALRGTYAGLGHPATIEHLKKVGVTAVELLPVHHIVDEPFLVERGLT.....NY
2WSK_A LHPEIPVEIRGTYKALGHPVMINYLKQLGITALELLPVAQFASEPRLQRMGLS.....NY
4J7R_A AHASSGVAAPGTYAGMVER..LDYLKSLGVNAIELLPVFEFNELEYYSQIPGSDQYRFNF
2VNC_A η6 α6 β13 TT T
250 260 270 280 290
2VNC_A WGYDPINFFSPECRYSSTG....CLGGQVLSFKKMVNELHNAGIEVIIDVVYNHTAEGNH
MCK8502360.1 WGYSTLGYFAPDARYSTAG....APGAQVDEFKGMVKALHRAGIEVILDVVYNHTCEGNQ
2WSK_A WGYNPVAMFALHPAYACS......PETALDEFRDAIKALHKAGIEVILDIVLNHSAELDL
4J7R_A WGYSTVNYFSPMGRFSAAVGQGAPARASCDEFKQLVKECHRRGIEVILDVVFNHTAEGNE
1 1
2VNC_A T α7 TTT TT TT α8
300 310 320 330 340 350
2VNC_A LGPTLSFRGIDNTAYYMLQPDNKRYYLDFTGTGNTLNLSHPRVIQMVLDSLRYWVTEMHV
MCK8502360.1 LGPTLSFKGLDNGAYYRLTEKDPRYYLDVTGTGNSWNATHPYALKLVADSLRYWVEEMHV
2WSK_A DGPLFSLRGIDNRSYYWIREDGD..YHNWTGCGNTLNLSHPAVVDYASACLRYWVETCHV
4J7R_A RGPTISFRGLDNRVYYMLAPGGE..YYNYSGCGNTLNCNQPVVRQFILDCLKHWVTEYHV
2VNC_A β14 α9
360 370
2VNC_A DGFRFDLAAALARELYSVN.......................................ML
MCK8502360.1 DGFRFDLATTLGRDRHGYD.......................................TR
2WSK_A DGFRFDLAAVMGR.TPEFR.......................................QD
4J7R_A DGFRFDLASILTRAHSAWHPQQYDQETGQRVAMSSGGAIVTAEGIMTDGAGVPTGYPLAD
2VNC_A α10 β15 TT β16 α11
380 390 400 410 420 430
2VNC_A NTFFIALQQDPILSQVKLIAEPWDVGQGGYQVGNFPYQ...WAEWNGKYRDSIRRFWRGE
MCK8502360.1 AAFFQIIHQDPVLSRVKLISEPWDVGDFGYQVGNFPVL...WSEWNGKYRDTIRRYWKGD
2WSK_A APLFTAIQNCPVLSQVKLIAEPWDIAPGGYQVGNFPPL...FAEWNDHFRDAARRFWLHY
4J7R_A PPLVESISEDPVLRNTKMIAEAWDC.DGLNQVGAFPHYGGRWSEWNGKFRDVVRNFIKGT
2VNC_A β17 α12 η7 β18 α13
440 450 460 470
2VNC_A ALPYS.EIANRLLGSPDIYLGN..................NKTPFASINYVTSHDGFTLE
MCK8502360.1 DRQAA.EIGYRLTGSSDLYSLS..................GRKPSASVNFVTAHDGFTLH
2WSK_A DLPLG.AFAGRFAASSDVFKRN..................GRLPSAAINLVTAHDGFTLR
4J7R_A DGPWAGDFASAICGSPNIYANNTPHETDWWANNGGRQWKGGRGPHASINFVAAHDGFTLA

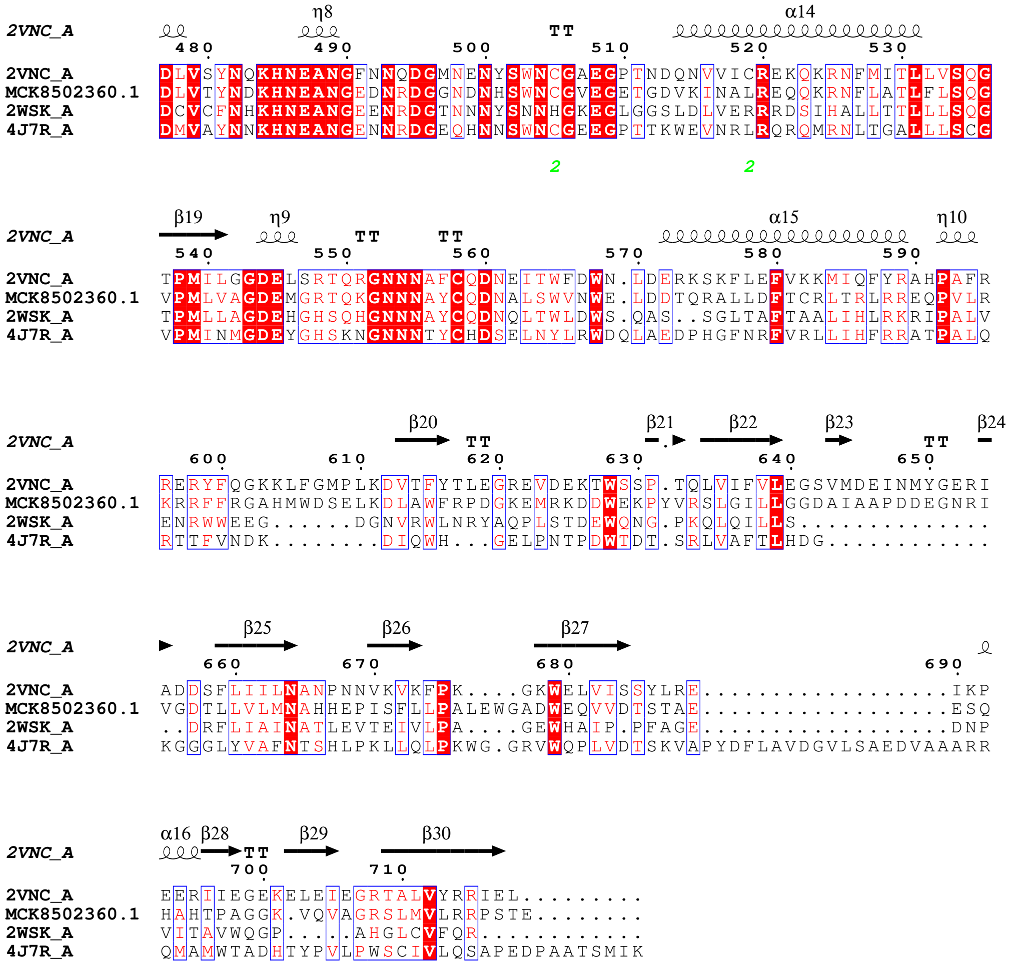

2VNC_A η8 α14 TT
480 490 500 510 520 530
2VNC_A DLVSYNQKHNEANGFNNQDGMNENYSWNCGAEGPTNDQNVVICREKQKRNFMITLLVSQG
MCK8502360.1 DLVTYNDKHNEANGEDNRDGGNDNHSWNCGVEGETGDVKINALREQQKRNFLATLFLSQG
2WSK_A DCVCFNHKHNEANGEENRDGTNNNYSNNHGKEGLGGSLDLVERRRDSIHALLTTLLLSQG
4J7R_A DMVAYNNKHNEANGENNRDGEQHNNSWNCGEEGPTTKWEVNRLRQRQMRNLTGALLLSCG
2 2
2VNC_A β19 η9 TT TT α15 η10
540 550 560 570 580 590
2VNC_A TPMILGGDELSRTQRGNNNAFCQDNEITWFDWN.LDERKSKFLEFVKKMIQFYRAHPAFR
MCK8502360.1 VPMLVAGDEMGRTQKGNNNAYCQDNALSWVNWE.LDDTQRALLDFTCRLTRLRREQPVLR
2WSK_A TPMLLAGDEHGHSQHGNNNAYCQDNQLTWLDWS.QAS..SGLTAFTAALIHLRKRIPALV
4J7R_A VPMINMGDEYGHSKNGNNNTYCHDSELNYLRWDQLAEDPHGFNRFVRLLIHFRRATPALQ
2VNC_A β20 TT β21 β22 β23 TT β24
600 610 620 630 640 650
2VNC_A RERYFQGKKLFGMPLKDVTFYTLEGREVDEKTWSSP.TQLVIFVLEGSVMDEINMYGERI
MCK8502360.1 KRRFFRGAHMWDSELKDLAWFRPDGKEMRKDDWEKPYVRSLGILLGGDAIAAPDDEGNRI
2WSK_A ENRWWEEG......DGNVRWLNRYAQPLSTDEWQNG.PKQLQILLS..............
4J7R_A RTTFVNDK........DIQWH...GELPNTPDWTDT.SRLVAFTLHDG............
2VNC_A β25 β26 β27
660 670 680 690
2VNC_A ADDSFLIILNANPNNVKVKFPK....GKWELVISSYLRE..................IKP
MCK8502360.1 VGDTLLVLMNAHHEPISFLLPALEWGADWEQVVDTSTAE..................ESQ
2WSK_A ..DRFLIAINATLEVTEIVLPA....GEWHAIP.PFAGE..................DNP
4J7R_A KGGGLYVAFNTSHLPKLLQLPKWG.GRVWQPLVDTSKVAPYDFLAVDGVLSAEDVAAARR
2VNC_A α16 β28 TT β29 β30
700 710
2VNC_A EERIIEGEKELEIEGRTALVYRRIEL.........
MCK8502360.1 HAHTPAGGK.VQVAGRSLMVLRRPSTE........
2WSK_A VITAVWQGP.....AHGLCVFQR............
4J7R_A QMAMWTADHTYPVLPWSCIVLQSAPEDPAATSMIK
