# Supplementary material for: Molecular Cloning, Characterization, and Application of a Novel Multifunctional Isoamylase (MIsA) from Myxococcus sp. Strain V11
Source: Foods. 2024 Oct 30;13(21):3481. doi: 10.3390/foods13213481 (PMC11544908; doi:10.3390/foods13213481)
Supplement: Supplementary file 1 [file foods-13-03481-s001.zip › Table S1.pdf]

Table S1. Yield analysis of recombinant starch debranching enzymes for hydrolysis of amylopectin and amylose $^{\alpha}$

Substrate	Starch input (mg)	Reducing sugar production (mg)	Yield %±SD
Amylose	2.5	0.254	10.158 ±0.061[b]
Amylopectin	2.5	0.293	11.703 ±0.062[a]
