# Supplementary material for: Molecular Cloning, Characterization, and Application of a Novel Multifunctional Isoamylase (MIsA) from Myxococcus sp. Strain V11
Source: Foods. 2024 Oct 30;13(21):3481. doi: 10.3390/foods13213481 (PMC11544908; doi:10.3390/foods13213481)
Supplement: Supplementary file 1 [file foods-13-03481-s001.zip › Table S2.pdf]

Table S2. Site-directed Mutagenesis of the Active Site of MIsA

Site	Substrates	Activity (%)
D352	Amylopectin	0
	Amylose	0
E388	Amylopectin	0
	Amylose	0
D460	Amylopectin	0
	Amylose	0

Active Site of MIsA for hydrolysis of amylopectin and amylose, and was incubate d in Na_2HPO_4-NaH_2PO_4 buffer (50 mM; pH 6.0) for 0.5 h at 50°C with different s ubstrates (0. 5%, wt/vol).
