# Supplementary figures and images for: Molecular Cloning, Characterization, and Application of a Novel Multifunctional Isoamylase (MIsA) from Myxococcus sp. Strain V11
Source: Foods. 2024 Oct 30;13(21):3481. doi: 10.3390/foods13213481 (PMC11544908; doi:10.3390/foods13213481)

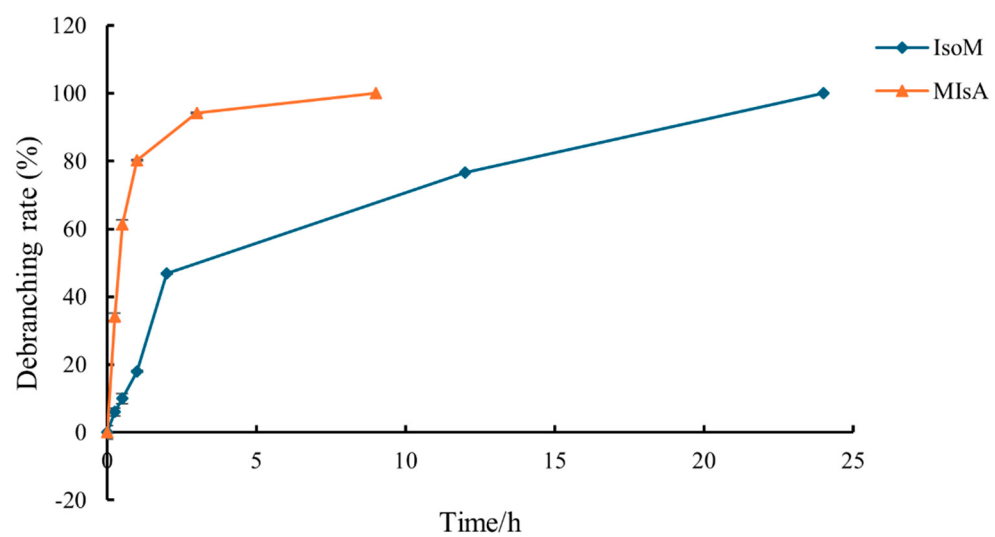

Figure S2. Comparison of MIsA and IsoM Degradation.

Supplement: Supplementary file 1 [file foods-13-03481-s001.zip › Fig.S2.pdf]

# G1

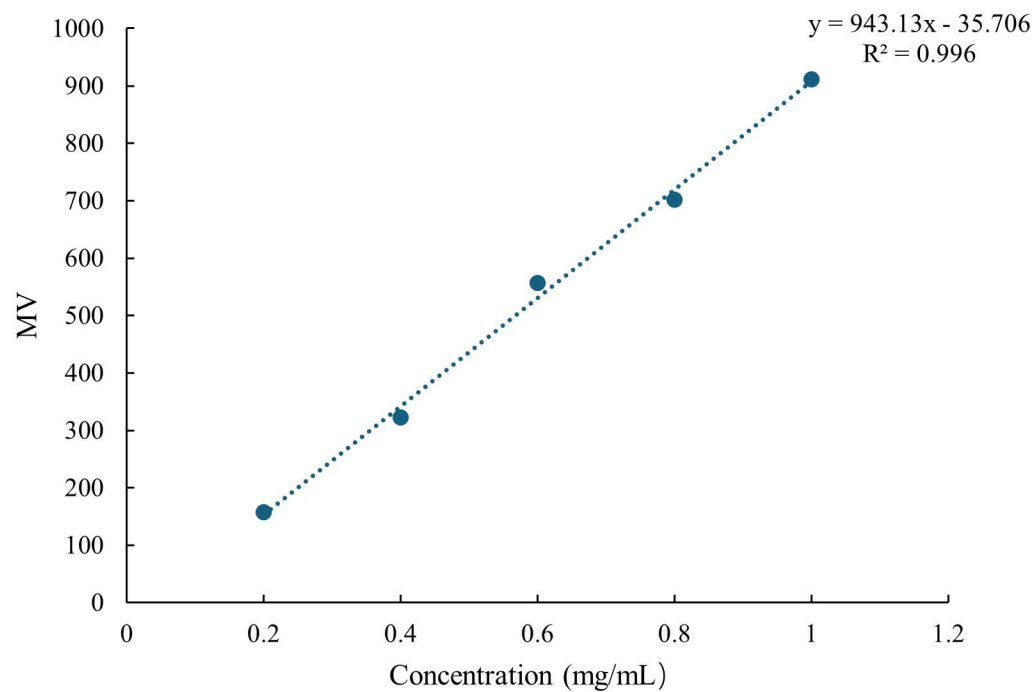

# G2

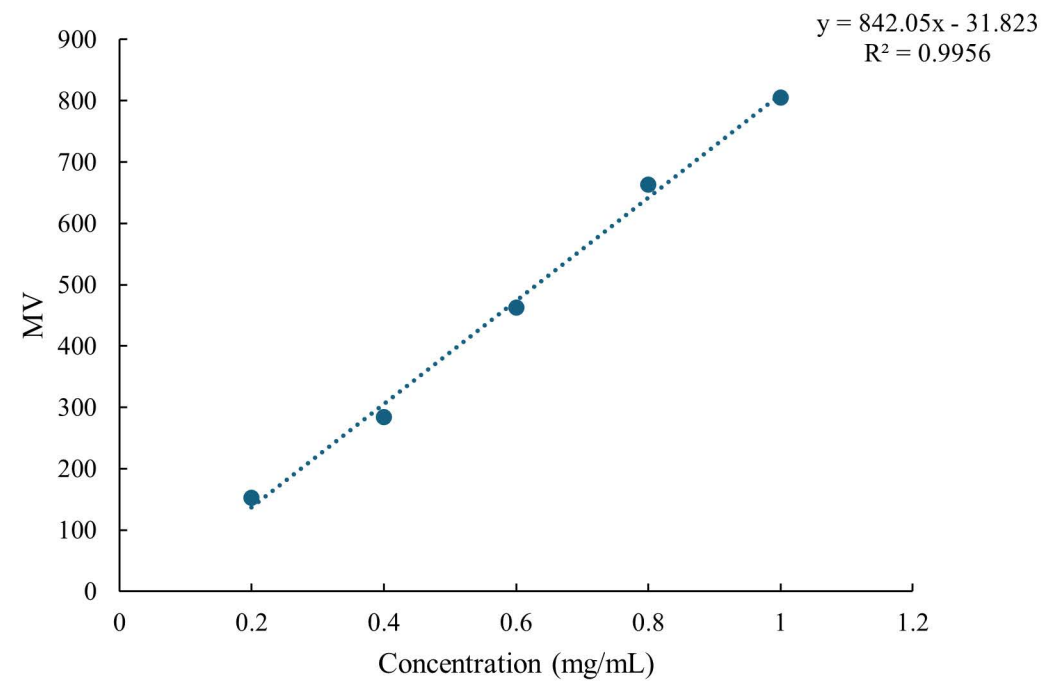

# G3

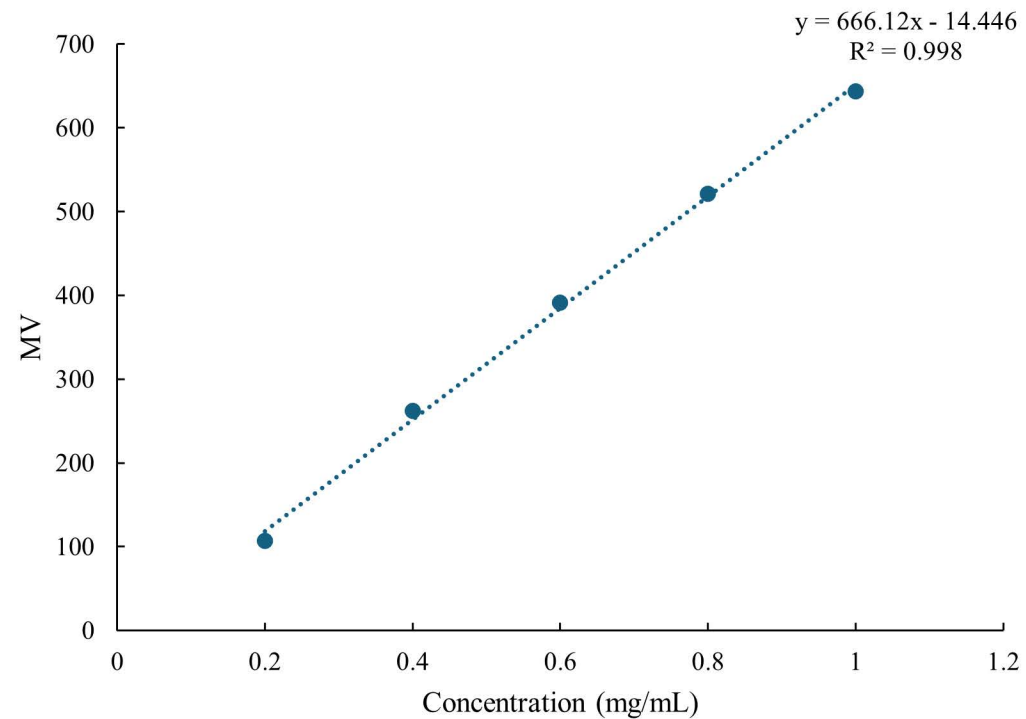

# G4

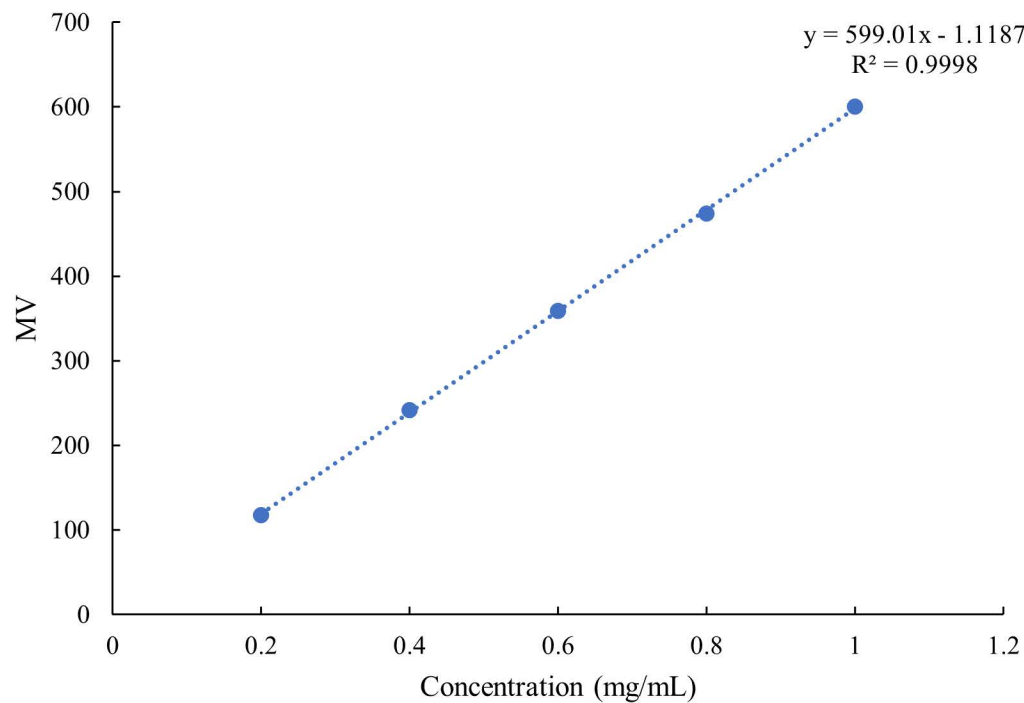

Supplement: Supplementary file 1 [file foods-13-03481-s001.zip › Standard Curves for G1ú¿aú⌐íóG2ú¿bú⌐íóG3ú¿cú⌐íóG4ú¿dú⌐.tif.pdf]
